# Editor's Letter-Box

**Published:** 1898-02-05

**Authors:** 


### Editor's Letter-Box.

[Our Correspondents are reminded that prolixity is a great bar to publication, and that brevity of style and conciseness of statement greatly facilitate early insertion.]

Mr. M. C. Seton, who has recently returned from South Africa, writes urging us to insist yet again on the folly of moribund and pauper patients going to the Cape. He says he has seen in Cape Colony numbers of poverty-stricken consumptives who ought never to have come out gradually dying of want, and he describes how wretched is the fate of those who are too ill to do work and yet too poor to live without it. It is a matter for very careful consideration. There are places nearer home where, at least, patients can meet their fate in moderately comfortable surroundings; but in South Africa, unless they get well, they die very miserably indeed.

